# Machine learning-based identification of SOX10 as an immune regulator of macrophage in gliomas

**DOI:** 10.3389/fimmu.2022.1007461

**Published:** 2022-11-29

**Authors:** Gelei Xiao, Kaiyue Wang, Zeyu Wang, Ziyu Dai, Xisong Liang, Weijie Ye, Peng Luo, Jian Zhang, Zaoqu Liu, Quan Cheng, Renjun Peng

**Affiliations:** ^1^ Department of Neurosurgery, Xiangya Hospital, Central South University, Changsha, China; ^2^ Diagnosis and Treatment Center for Hydrocephalus, Xiangya Hospital, Central South University, Changsha, China; ^3^ Hunan International Scientific and Technological Cooperation Base of Brain Tumor Research, Xiangya Hospital, Central South University, Changsha, China; ^4^ National Clinical Research Center for Geriatric Disorders, Xiangya Hospital, Central South University, Changsha, China; ^5^ Xiangya School of Medicine, Central South University, Changsha, China; ^6^ MRC Centre for Regenerative Medicine, Institute for Regeneration and Repair, University of Edinburgh, Edinburgh, United Kingdom; ^7^ Department of Clinical Pharmacology, Xiangya Hospital, Central South University, Changsha, China; ^8^ Department of Oncology, Zhujiang Hospital, Southern Medical University, Guangzhou, China; ^9^ Department of Interventional Radiology, The First Affiliated Hospital of Zhengzhou University, Zhengzhou, China

**Keywords:** gliomas, SOX10, immunotherapy, immune infiltration, immune microenvironment

## Abstract

Gliomas, originating from the glial cells, are the most lethal type of primary tumors in the central nervous system. Standard treatments like surgery have not significantly improved the prognosis of glioblastoma patients. Recently, immune therapy has become a novel and effective option. As a conserved group of transcriptional regulators, the Sry-type HMG box (SOX) family has been proved to have a correlation with numerous diseases. Based on the large-scale machine learning, we found that the SOX family, with significant immune characteristics and genomic profiles, can be divided into two distinct clusters in gliomas, among which SOX10 was identified as an excellent immune regulator of macrophage in gliomas. The high expression of SOX10 is related to a shorter OS in LGG, HGG, and pan-cancer groups but benefited from the immunotherapy. It turned out in single-cell sequencing that SOX10 is high in neurons, M1 macrophages, and neural stem cells. Also, macrophages are found to be elevated in the SOX10 high-expression group. SOX10 has a positive correlation with macrophage cytokine production and negative regulation of macrophages’ chemotaxis and migration. In conclusion, our study demonstrates the outstanding cluster ability of the SOX family, indicating that SOX10 is an immune regulator of macrophage in gliomas, which can be an effective target for glioma immunotherapy.

## Introduction

Gliomas, originating from the glial cells, are the most lethal type of primary tumors in the central nervous system (CNS) (1). According to the WHO classification criteria, they are classified into four types inferred by malignancy (2). In depth, grade II and III gliomas are classified as lower-grade gliomas (LGG), grade IV (glioblastoma, GBM) as higher-grade gliomas (HGG), by The Cancer Genome Atlas (TCGA). For most cases, LGG with the isocitrate dehydrogenase (IDH) mutant for the metabolic enzymes has a conspicuously better prognosis than the IDH wild type, which are generally GBMs. To date, the standard treatment contains surgery has not significantly improved the prognosis and median overall survival (OS) of GBM patients (3). As a consequence, a new and effective therapy is of urgency.

Recent studies have found that as a constitutive part of the tumor microenvironment (TME), tumor cells, stromal cells, and infiltrating immune cells all serve a variety of biologically important roles in glioma proliferation, progression, and prognosis (4). Moreover, we and others have previously suggested several immune-related prognostic biomarkers to predict prognosis and immunotherapy efficacy perfectly (5, 6). These may all contribute to the immune therapy of glioma.

Sry-type HMG box (SOX) family proteins are a conserved group of transcriptional regulators depending on the high-mobility group (HMG) domain to bind with DNA (7). The SOX family has been revealed to have the correlation with numerous diseases (8). Almost all SOX genes, for instance, SOX1, SOX2, SOX7, and SOX10, have been found to have the potential to regulate the progression of glioma, whose expression levels are also related to the prognosis of patients (9–12). SOX genes play an important role in this regulation, which are found to be involved in the maintenance of the stemness or differential initiation of glioma stem cells (13). For example, knockdown of SOX1 expression in glioma stem cells has been found to impair the self-renewal, proliferation, viability, and tumorigenesis ability of glioma cells, while the overexpression of SOX1 promoted the malignant phenotype of glioma (9). However, the overexpression of SOX11 prevents tumorigenic ability in glioma-initiating cell-like cells and human glioma-initiating cells derived from malignant gliomas by inducing neuronal differentiation (14). Moreover, previous studies have confirmed that SOX is closely associated with the TME (15). SOX genes in tumor cells influence the infiltration of immune cells *via* paracrine signals, and vice versa (16). By giving tumor cells the ability to evade NK cells, SOX2 and SOX9 have been found to promote the immune evasion of tumor cells (17, 18). Therefore, the SOX family is crucial to the development of gliomas, especially in the aspect of the TME and immunotherapy. However, it remains unclear which one, as well as the detailed function, of the SOX family plays the leading role in glioma.

Herein, our study extracted data from bulk tumor (The Cancer Genome Atlas, TCGA; the Chinese Glioma Genome Atlas, CGGA) and single-cell mRNA-seq databases (SCP50 and SCP393; http://singlecell.broadinstitute.org). Cluster analysis was performed, and SOX10 was identified as a distinguished biomarker to explore the prognostic value and association with the glioma immune microenvironment.

## Materials and Methods

### Data collection and preprocessing

1685 samples of diffuse glioma related data and complete clinicopathological annotations were obtained from two datasets: TCGA (https://xenabrowser.net/) and CGGA (http://www.cgga.org.cn/). 672 samples in TCGA were used as the training set, while 1013 samples in CGGA were used as the validation set. We excluded samples with insufficient OS. The RNA-sequencing data, SCP50 and SCP393, was collected form Single Cell Portal platform (http://singlecell.broadinstitute.org). To possessing a similar signal intensity with the RMA- processed values, the fragments per kilobase million (FPKM) values were transformed into transcripts per kilobase million (TPM) values.

### Genomic alteration

We obtained the somatic mutation and copy number variant (CNV) profiles from TCGA dataset. We used GISTIC 2.0 analysis (https://cloud.genepattern.org) to assess the landscape of CNV, including the frequency of function mutation gain or loss at the amplified or deleted peaks.

### Unsupervised consensus clustering for the SOX family and the selection of SOX10

Using the ConsensusClusterPlus R package, we determined the optimal cluster number and their constancy and authenticity in TCGA cohort and meta-cohort. We performed principal component analysis (PCA) to ensure the clustering tendency. The LASSO-LR algorithm, Pamr algorithm, random forest algorithm, XGboost algorithm, and Boruta algorithm were used to screen out the most characteristic genes, SOX10.

### TME immunological characteristic analysis

The Estimation of STromal and Immune cells in MAlignant Tumors using Expression data (ESTIMATE) algorithm was used to estimate the stromal score, immune score, and estimate score of the infiltrating immune cells in the TME. The Tumor Immune Estimation Resource 2.0 (TIMER2.0; http://timer.cistrome.org/) web server was used to thoroughly evaluate the degree of immune infiltrating cells in gliomas. We used the xCell algorithm to ascertain the enrichment levels of 64 types of immune cells. The proportions of 22 types of TME cells in tumor tissues were evaluated by the CIBERSORT algorithm. The R gene set variation analysis (GSVA) package was implemented to calculate enrichment scores by single-sample gene set enrichment analysis (ssGSEA). Besides, the EPIC algorithm, MCPcounter algorithm, and QuantSeq algorithm were also executed to estimate the immune infiltrating cell abundance. Using ssGSEA, we evaluated the seven steps of cancer immune cascades. This immunity cycle determined the destination of tumor cells and reflected the immune response of the anticancer. The subMap algorithm was used to evaluate the response to therapies of anti-CTLA4 and anti-PD1. Also, the Tumor Immune Dysfunction and Exclusion (TIDE) (http://tide.dfci.harvard.edu/setquery/) and Tumor Immune Syngeneic MOuse (TISMO) (http://tismo.cistrome.org) algorithm was utilized for deducing the immune checkpoint blockade immunotherapy responses in gliomas.

### Single-cell sequencing

R package Seurat was employed to process the single-cell data expression matrix. We used “NormalizeData” to renormalize data. Then, 2,000 highly changeable genes were identified by “FindIntegrationAnchors.” “FindIntegrationAnchors” and the “Intergratedata” were used to merge GBM sample data sets. “RunPCA” and “FindNeighbors” was used to perform PCA. Afterwards, to alternately combine cells together, we used the “FindClusters” function. Finally, to visualize the analyses, “UMAP” was performed.

### Multiplex immunofluorescence staining

We purchased the glioma tissue array from Wuhan Tanda Scientific Co., Ltd. (NGL1021), with ethics approvement. SOX10 (Mouse, 1:100, Proteintech, China), CD163 (Rabbit, 1:3,000, Proteintech, China), and CD68 (Rabbit, 1:3,000, Servicebio, China) were the primary Abs. Horseradish peroxidase-conjugated secondary antibody incubation (GB23301, GB23303, Servicebio, China) was the secondary antibody. The tyramide signal was amplified into TSA [FITC-TSA, CY3-TSA, 594-TSA, and 647-TSA (Servicebio, China)]. The stained slides were scanned using the TissueFAXS platform (TissueGnostics, Vienna, Austria). The spatial analysis of the stained cells was performed using the StrataQuest software (TissueGnostics, Vienna, Austria).

### Drug response prognostication

All pharmacogenomic data were downloaded from the Genomics of Drug Sensitivity in Cancer (GDSC, https://www.cancerrxgene.org/). The semi-inhibition rate (IC50) reckoned by the pRRophetic R package was utilized to predict the drug susceptibilities and responses.

### Statistical analysis

The overall survival of divergent groups was assessed by Kaplan–Meier curves (KM curves) with the log-rank test. All OS curves were produced by the survminer R package. Mutation landscape OncoPrint was executed by the maftools R package. Heatmaps were pictured found on the R package complexHeatmap. Student’s t-test was conducted to analysis normally distributed variables between the two groups while one-way analysis of variance (ANOVA) was conducted to analysis normally distributed variables between multiple groups. The Wilcoxon test was applied to analysis non-normally distributed variables between the two groups while Kruskal-Wallis test was applied to analysis normally distributed variables between multiple groups. R 3.6.3 was used to conduct all statistical analyses. Statistics were considered significant when p-value< 0.05.

## Results

### Two distinct clusters of the SOX family

**Figure 1:**
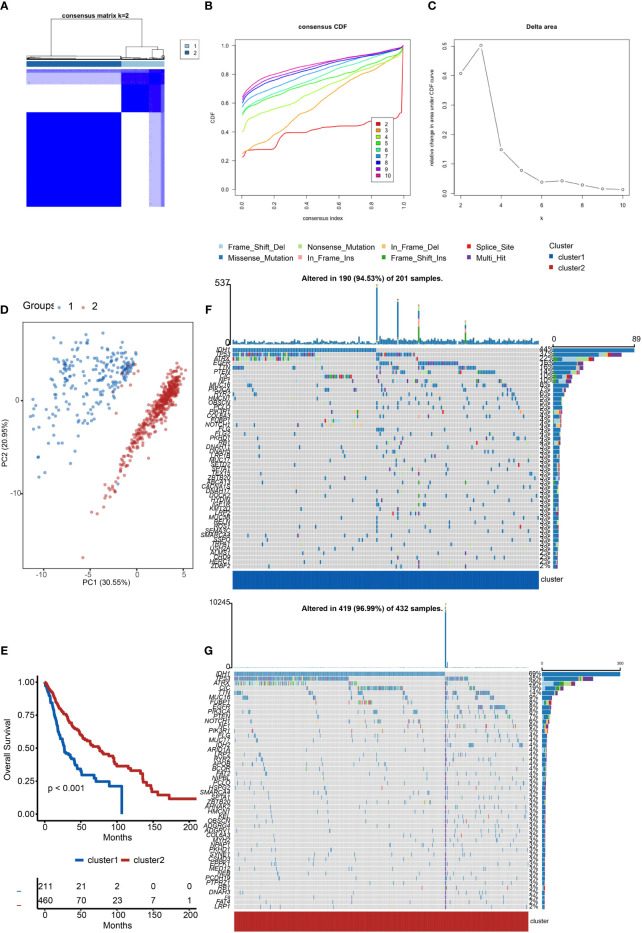
Cluster analysis of the SOX family. **(A)** Visualization of cluster analysis. **(B, C)** Determination of the number of clusters. **(D)** Significant separation of SOX clusters by PCA. Blue dots represent cluster1, while red dots represent cluster2. **(E)** Kaplan–Meier overall survival curves comparing cluster1 and cluster2 in gliomas. **(F)** Detection of the genes with the highest mutation frequency in cluster1. **(G)** Detection of the genes with the highest mutation frequency in cluster2.

Firstly, we evaluated the clustering capabilities of the SOX family and visualized it **(** **Figure 1A** **)**. To choose the ideal cluster number, the stability of clustering was appraised by the ConsensusClusterPlus package in TCGA **(** **Figures 1B, C** **)**. It was found that k = 2, with the flattest CDF curve, is the optimal choice **(** **Figure 1B** **)**. Then, clustering tendency was evaluated by principal component analysis (PCA). We used blue dots to represent cluster1, while red dots represent cluster2. SOX clusters were separated significantly, indicating a high-quality consensus cluster result **(** **Figure 1D** **)**.

We further explored the overall survival of glioma patients in cluster1 and cluster2, p < 0.001. The Kaplan–Meier curves firmly demonstrated that cluster2 had higher and more prolonged survival than cluster1 **(** **Figure 1E** **)**. Besides, **Figures 1F, G** show the global view of mutational distribution in cluster1 and cluster2, respectively. As a biomarker related to the malignancy of gliomas, IDH1 mutation took up 69% of the general in cluster2, much higher than that of cluster1, 44% (19, 20). Cellular tumor antigen p53 (TP53) alteration was presented similarly in cluster1 (37%) and cluster2 (45%). In cluster1, the following three genes ranked by frequency were alpha-thalassemia/mental retardation syndrome x-linked chromatin remodeler (ATRX) (22%), epidermal growth factor receptor (EGFR) (16%), and titin (TTN) (16%), while those in cluster2 were ATRX (29%), CIC (19%), and TTN (14%). In conclusion, the SOX family has a close correlation with the prognosis and proliferation of gliomas.

### Immune characteristics of two clusters

**Figure 2:**
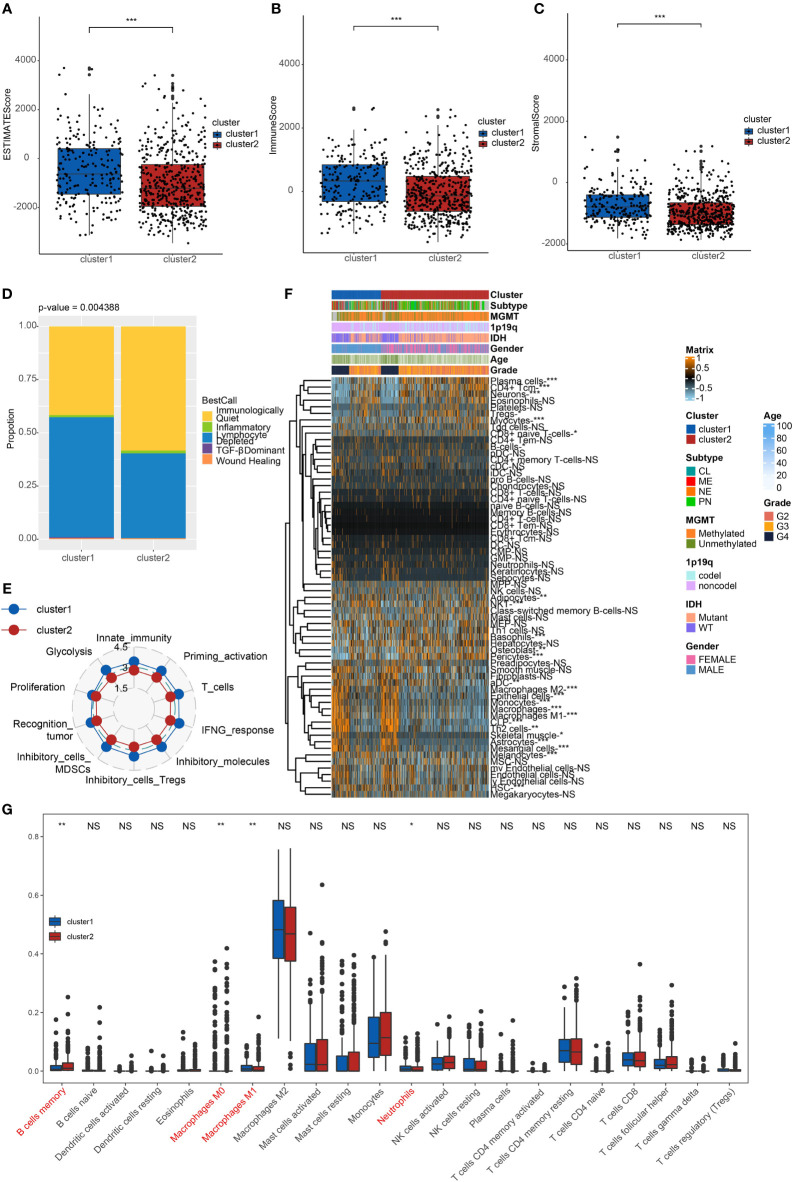
Immune characteristics of the clusters. **(A)** ESTIMATEScore **(B)** ImmuneScore. **(C)** StromalScore of cluster1 and cluster2 in TCGA. ***p < 0.001. **(D)** The proportion of five immune subtypes in cluster1 and cluster2. p = 0.004388. **(E)** The tendency of the difference between two clusters based on TME indicator scores. **(F)** Dendrogram corresponding to the 64-cell type level calculated by xCell and clusters in TCGA. *p < 0.05, **p < 0.01, ***p < 0.001. **(G)** Box plots of the proportions of 22 TME cell types in tumor tissues with cluster1 and cluster2. The dispersed dots represent values of TME cell expression in each cluster. **p < 0.01, *p < 0.05, NS, no significance.

We investigated the TME characteristics of cluster1 and cluster2. We evaluated the ESTIMATEScore, ImmuneScore, and StromalScore of the two clusters in TCGA dataset **(** **Figures 2A–C** **)**. Among these three evaluations, scores of cluster1 were all higher than those of cluster2. Moreover, they could be thought to have a significant difference, on account of p < 0.001. Then, we calculated the proportion of five immune subtypes in the two clusters **(** **Figure 2D** **)**. Immunologically Quiet was generally presented in cluster2 (more than 50%) and was partially observed in cluster1 (less than 50%). On the contrary, Lymphocyte Depleted was the frequentist immune subtype in cluster1, which took up over 50%. Moreover, we found that the TME indicator scores of cluster2 seemed to be lower than those of cluster1, which indicated a weaker immune response **(** **Figure 2E** **)**. It also revealed the difference between the two clusters.

We calculated relating levels of 64 cell types by the xCell algorithm and clusters in TCGA **(** **Figure 2F** **)**. We defined four subtypes of glioma: pro-neural (PN), classical (CL), neural (NE), and mesenchymal (ME), among which CL and ME are more severe (21). It is found that some types of cells are different in the two clusters with statistical significance. Plasma cells and neurons are more positively related to cluster2, while macrophages, macrophages M1, and macrophages M2 are more positively related to cluster1. Additionally, we used box plots to present the proportions of 22 TME cell types in tumor tissues with cluster1 and cluster2 **(** **Figure 2G** **)**. Only four cell types had significant differences: B cells memory, macrophages M0, M1, and neutrophils. B cells memory in cluster2 were higher than in cluster1. Meanwhile, macrophages M1 and neutrophils in cluster2 were lower than in cluster1.

### Distinct genomic profiles of the two clusters

**Figure 3:**
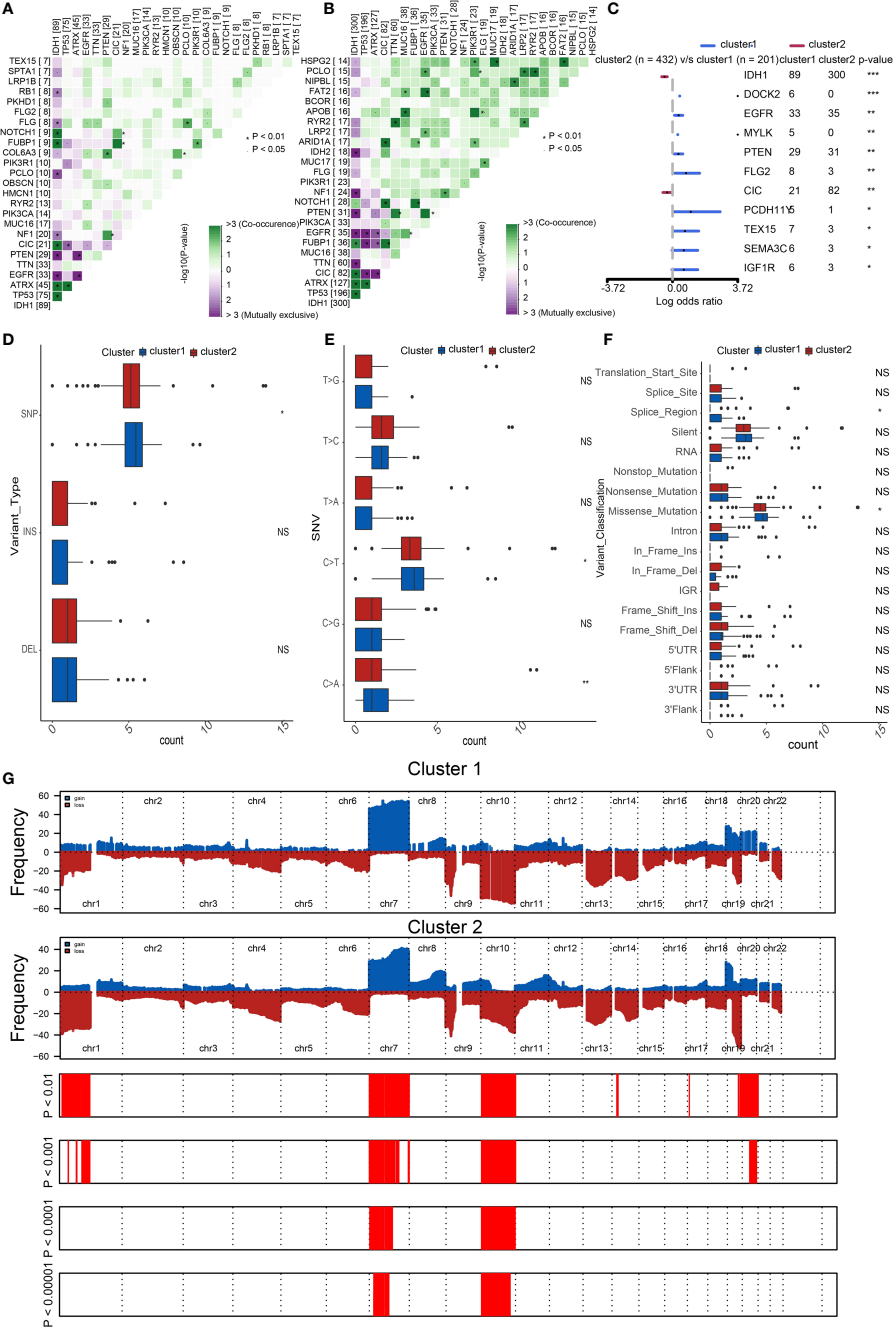
Distinct genomic profiles of the two clusters. The co-occurrence or mutual exclusivity of the top 25 most mutated genes in **(A)** cluster1 and **(B)** cluster2. *p < 0.01, `p < 0.05. **(C)** Demonstration of the 11 most altered genes between the clusters by the forest plot. Frequency comparison according to **(D)** variant type, **(E)** SNV, and **(F)** variant classification between the two clusters. **(G)** Amplifications and deletions in two clusters of SOX family by GISTIC 2.0. ***p < 0.001, **p < 0.01, *p < 0.05, NS, no significance.

Considering the apparent differences in overall survival and immune characteristics in cluster1 and cluster2, genomic profiles of the two clusters were supposed to be distinct. To validate it, we analyzed the co-occurrence/mutual exclusivity of the 25 most altered genes in cluster1 **(** **Figure 3A** **)** and cluster2 **(** **Figure 3B** **)**. The strongest co-occurrent couples of gene mutation in cluster1 and cluster2 were IDH1 and ATRX, IDH1 and CIC, IDH1 and FUBP1, ATRX and TP53. IDH1 and EGFR were mutually exclusive pairs in cluster1 and cluster2. Higher co-occurrence is usually functionally linked to the proliferation of gliomas (22, 23). Then, we used a forest plot to list the 11 most variated genes between the two clusters **(** **Figure 3C** **)**. Except for IDH1 and CIC, the other nine genes were more likely to mutate in cluster1.

Furthermore, we compared the frequency of different somatic mutations between the two clusters, including the single-nucleotide polymorphism (SNP), single nucleotide variant (SNV), deletion, insertion, and intergenic region (IGR). The frequency of insertion and deletion seemed to be non-statistically different, while SNPs were a little more common in cluster1 (**Figure 3D**). Among the identified SNVs, C was more presumably to mutate to T, which was also the most common mutation in cluster1 (**Figure 3E**). Transformation of splice region and missense were more common in cluster1 than in cluster2 (**Figure 3F**) (24). Amplifications and deletions of chr7 and chr10 have statistically differences in cluster1 and cluster2 (**Figure 3G**).

### Identification of SOX10 as a prognostic gene

**Figure 4:**
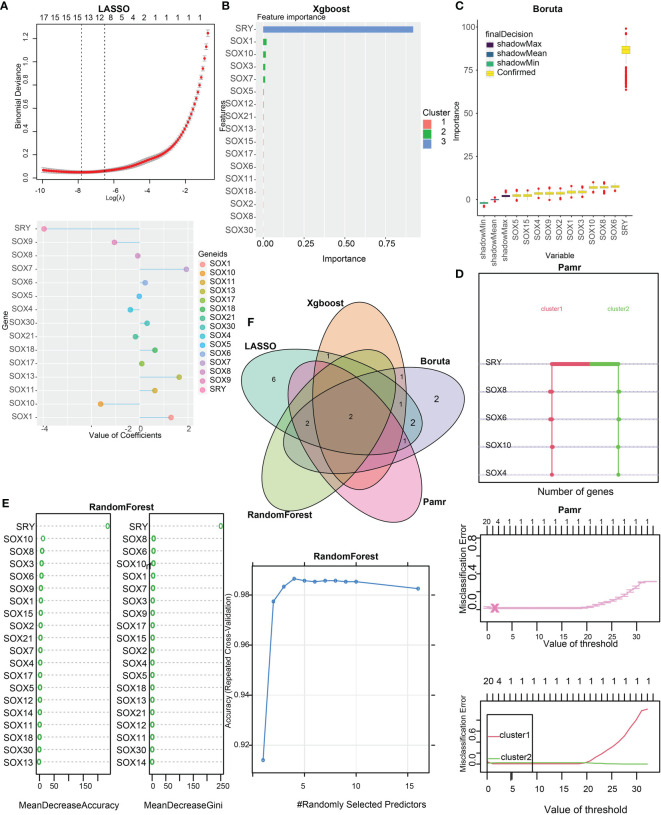
Identification of SOX10 as a prognostic gene by machine learning. **(A)** The assessment of the weighted importance of genes between two clusters by the LASSO-LR algorithm. **(B)** The evaluation of feature importance of genes between two clusters by the XGboost algorithm. **(C)** The selection of all relevant features of genes between two clusters by the Bruta algorithm. **(D)** The assessment of genes between two clusters by the Pamr algorithm. **(E)** The evaluation of genes between two clusters by the random forest algorithm. **(F)** Validation of the intersection of glioma prognostic genes from LASSO, Xgboost, Boruta, Pamr, and Random Forest.

To distinguish the two clusters more accurately and precisely, we executed machine learning and prediction on the two populations, screening out the most characteristic genes. Using the LASSO-LR, XGboost, Boruta, Pamr, and RandomForest machine learning algorithms, we filtrated 15, 5, 11, 5, and 4 genes, correspondingly **(** **Figures 4A–E** **)**. We used a Venn diagram to take the intersection of the five algorithms **(** **Figure 4F** **)**. These two characteristic genes in the intersection corner, SOX10 and sex determining region Y (SRY), were the most potential to best classify the two clusters (25). Considering that SRY mainly depends male sex, we identified SOX10 as a biomarker of glioma prognosis.

### The prognostic potential of SOX10

**Figure 5:**
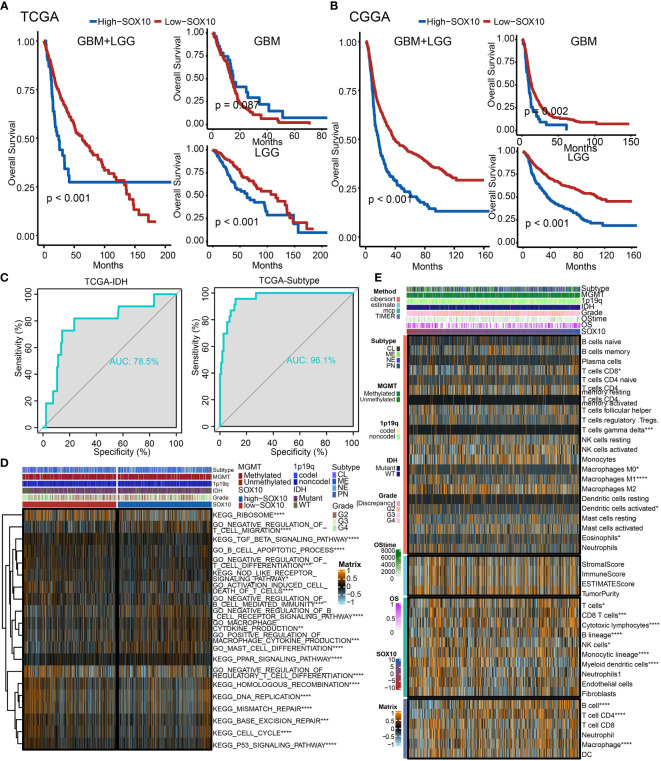
The prognostic potential of SOX10. Kaplan–Meier overall survival analysis of SOX10 in GBM, LGG, and pan-glioma based upon the **(A)** TCGA and **(B)** CGGA datasets. **(C)** Predictive value of SOX10, IDH, and subtype measured by ROC curves in TCGA dataset. **(D)** The heatmap for gene set variation analysis of SOX10 from TCGA. *p < 0.05, **p < 0.01, ***p < 0.001, ****p < 0.0001. **(E)** Heatmap visualized the abundance of infiltrating immune cell groups with divergent SOX10 degree. *p < 0.05, **p < 0.01, ***p < 0.001, ****p < 0.0001.

We performed a survival analysis of different SOX10 expressions in pan-glioma, LGG, and GBM based on TCGA and CGGA datasets **(** **Figures 5A, B** **)**. The Kaplan–Meier curves more securely demonstrated that grievous survival mischief in glioma patients with high SOX10. However, the GBM Kaplan–Meier curves in TCGA were contrary to those in CGGA, which could account for the small number of samples of GBM patients in TCGA. Moreover, we predicted the value of SOX10, IDH, and subtype measured by receiver operating characteristic (ROC) curves in TCGA dataset **(** **Figure 5C** **)**. The results firmly proved that SOX10 was a predictor of IDH and subtype. The ROC curves exhibited high sensitivity and specificity, with all areas under the curves (AUC) bigger than 0.7 and 0.9.

Additionally, to probe the latent pathological function of SOX10, the KEGG and GO enrichment analyses were performed. **Figure 5D** depicts 20 related pathways in the two selected pathways. The high expression of SOX10 seems to be correlated with the negative regulation of regulatory T-cell differentiation, DNA replication, and mismatch repair. Besides, **Figure 5E** demonstrates the abundance of infiltrating immune cell groups with divergent SOX10 expressions identified by the CIBERSORT, ESTIMATE, MCP, and TIMER algorithms of TCGA datasets. With the increasing expression of SOX10, the proportion of B cells, T cell CD4, and macrophages increased.

### SOX10 is associated with immunotherapy response

**Figure 6:**
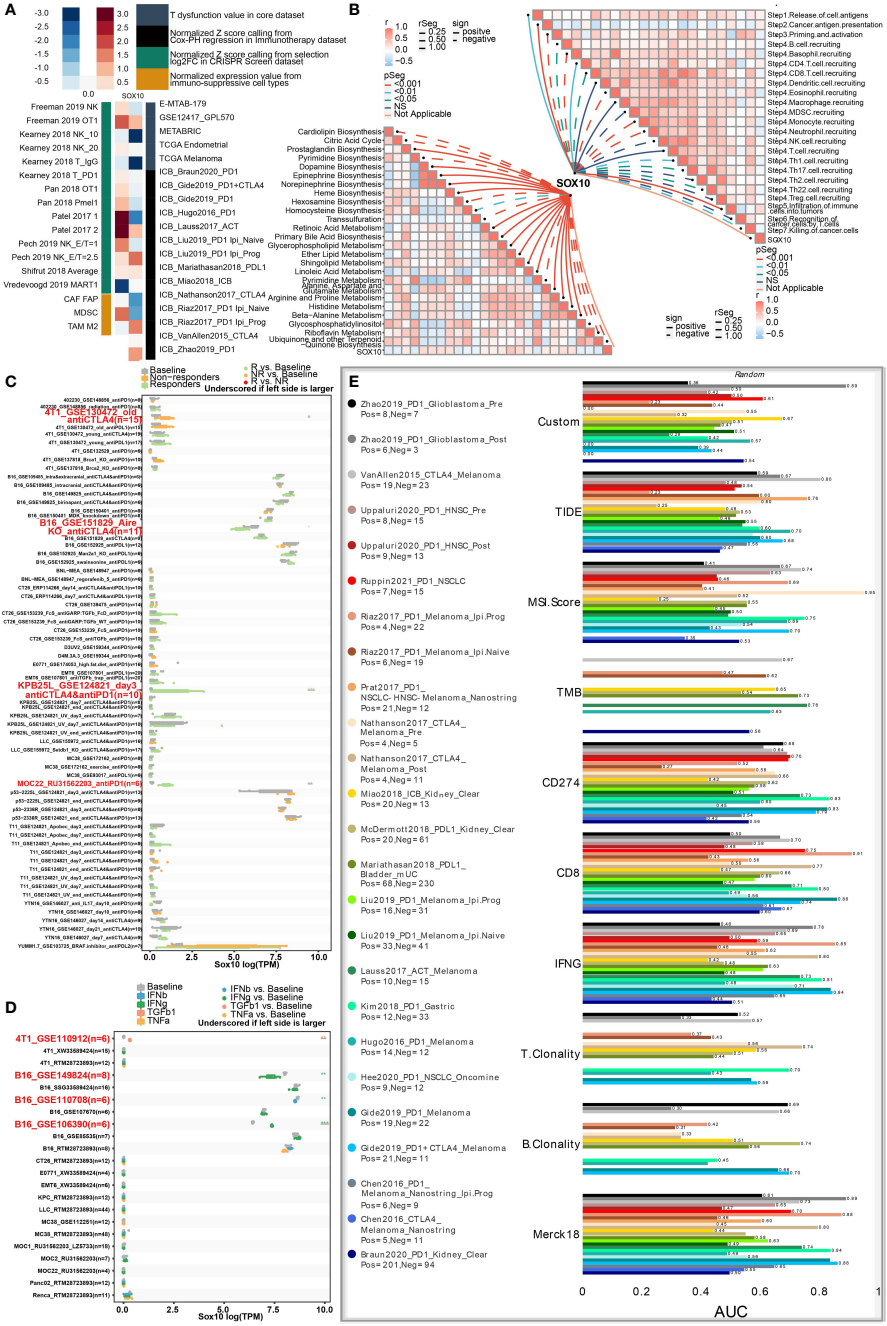
Roles of SOX10 in immunotherapy response, metabolism phenotypes, and biomarker relevance. **(A)** Heatmap showing the correlation with T-cell dysfunction, normalized Z score, and normalized expression. **(B)** Correlations between SOX10 and enrichment outcomes of metabolism-relevant pathways together with cancer-immune cascade steps. **(C)** Immunotherapy response of SOX10 in immunotherapy cohorts. **(D)** Immune effector molecule relevance of SOX10 in immunotherapy cohorts. **(E)** Biomarker relevance of SOX10 in immunotherapy cohorts.

As manifested in the heatmap **(** **Figure 6A** **)**, SOX10 was negatively correlated with T-cell dysfunction, implying its potential impact on immunotherapy. Specifically, SOX10 positively correlated with the normalized Z score from selection log2FC in the CRISPR screen dataset and normalized expression value from immune-suppressive cell types.

Metabolism has been considered a vital determining factor in the survivability and potency of immune cells (26). We explored correlations between SOX10 and enrichment scores of metabolism-pertinent pathways and cancer-immune cascade steps by GSVA. **Figure 6B** concludes that SOX10 was negatively associated with cardiolipin biosynthesis, citric acid cycle, trans-sulfuration, pyrimidine metabolism, ubiquinone, and another terpenoid. Notably, SOX10 was observed to be correlated with most steps of the immune cascade.

Then, to thoroughly analyze the prospective merit of SOX10 as a new immune target in pan-cancer, sensitive drugs predicated on SOX10 expression were predicted **(** **Figure S1A** **)**. We also explored the semi-inhibition rates of gefitinib and nilotinib. The results showed that the estimated IC50 was higher in low-expression SOX10 than in high-expression **(** **Figure S1B, C** **)**. Another noteworthy observation was that SOX10 could significantly predict immunotherapy response, whose responders were correlated with SOX10 levels **(** **Figure 6C** **)**. Besides, SOX10 could significantly predict the cytokine treatment of immune effector molecules in four immunotherapy cohorts **(** **Figure 6D** **)**. We also computed the biomarker pertinence of SOX10 by comparing it with normalized biomarkers based off of their prognosticative ability of response outcomes and OS of human immunotherapy cohorts. Fascinatingly, it was found that SOX10 gave an AUC of more than 0.5 in eight out of the 25 immunotherapy cohorts **(** **Figure 6E** **)**. SOX10 presented a better predictive value than B clonality, with AUC values over 0.5 in 8 immunotherapy cohorts. However, the prognosticative ability of SOX10 was lower than that of the TIDE (AUC > 0.5 in 18 immunotherapy cohorts), MSI score (AUC > 0.5 in 13 immunotherapy cohorts), TMB (AUC > 0.5 in 8 immunotherapy cohorts), CD274 (AUC > 0.5 in 21 immunotherapy cohorts), CD8 (AUC > 0.5 in 18 immunotherapy cohorts), IFNG (AUC > 0.5 in 17 immunotherapy cohorts), T clonality (AUC > 0.5 in 9 immunotherapy cohorts), and Merk 18 (AUC > 0.5 in 18 immunotherapy cohorts).

### Single-cell sequencing and SOX10 co-expression on glioma cells

**Figure 7:**
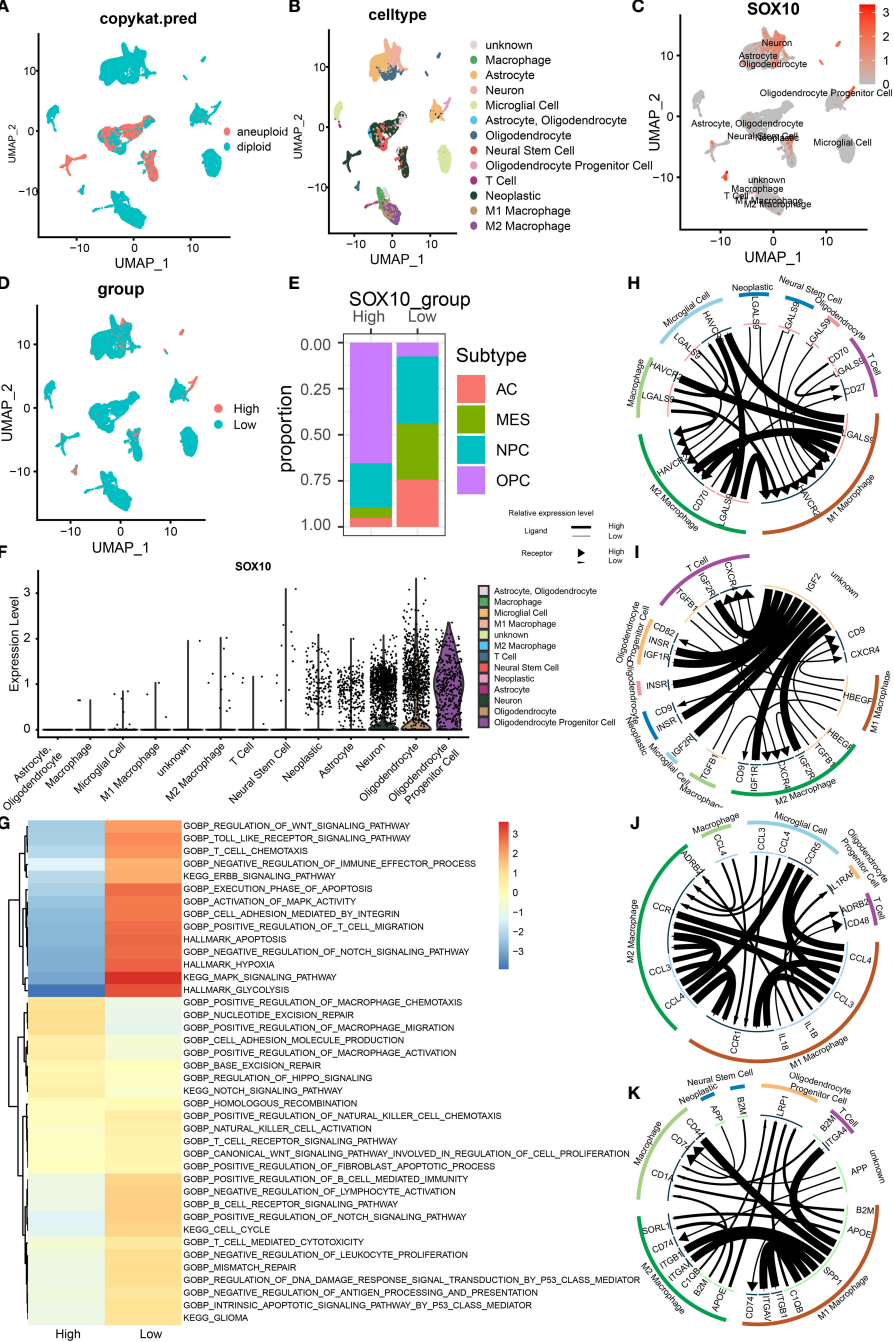
Stratification, identification, and SOX10 co-expression on glioma cells by single-cell sequencing analysis. **(A)** UMAP projection determined by CopyKat analysis. **(B)** UMAP projections of different cells, color-coded by cell types. **(C)** Annotation of different cell types and the expression of SOX10 in each cell type. **(D)** UMAP projections of two cell groups based on the expression of SOX10. **(E)** The proportion of glioma subtypes in the high and low SOX10 expression group. Astrocytes (AC), neural progenitor cells (NPC), mesenchyme (MES), and oligodendrocyte precursor cells (OPC). **(F)** Violin plot of SOX10 expression distribution of divergent cell clusters. **(G)** Enrichment analysis correlating divergent immune regulatory processes with high and low SOX10 expressions. **(H)** Correlation circles for positively and negatively correlated checkpoint genes in the high expression group of SOX10. **(I)** Correlation circles for positively and negatively correlated growth factors in the high expression group of SOX10. **(J)** Correlation circles for positively and negatively correlated cytokines in the high expression group of SOX10. **(K)** Correlation circles for positively and negatively correlated other genes in the high expression group of SOX10.

Finally, we utilized single-cell sequencing to analyze the circumstances of stratification, identification, and SOX10 co-expression on glioma cells. UMAP determined by Copynumber Karyotyping of Tumors analysis stratified cells into diploid (average) status and aneuploid (malignant) status **(** **Figure 7A** **)**. At the same time, we identified cell types and used UMAP to make it intuitionistic, which demonstrated 13 cell clusters **(** **Figure 7B** **)**. Similarly, the co-expression status of different types of cells is shown in **Figure 7C**. **Figure 7D** shows the division of cell clusters into two groups, based upon the high and low expression levels of SOX10. In the high-expression cluster of SOX10, OPC was observed to take more than 50% of all TME cells, followed by neuroprogenitor cells (NPC), mesenchyme (MES), and astrocyte (AC) **(** **Figure 7E** **)**. In descending order, the proportion of subtypes in a low-expression cluster of SOX10 was NPC (less than 50%), MES, AC, and OPC. The expression level of SOX10 in different subtypes is shown in the violin plot **(** **Figure 7F** **)**. It was found that SOX10 had a high expression in neoplastic cells, astrocytes, neurons, oligodendrocytes and oligodendrocyte progenitor cells.

Subsequently, we performed enrichment analysis to determine the correlation between different immune regulatory processes and SOX10 expressions. High-expression SOX10 was significantly positively associated with the Notch signaling pathway and migration and activation regulation of the macrophage. In contrast, low-expression SOX10 was positively associated with the activation of MAPK activity, MAPK signaling pathway, and regulation of T-cell migration **(** **Figure 7G** **)**. Moreover, we drew correlation circles for positively and negatively correlated checkpoint genes, growth factors, cytokines, and other genes in the SOX10 high-expression group **(** **Figures 7H–K** **)**. For checkpoint genes, we could see a strong correlation in LGALSS of M1 macrophages and HAVCR2 of microglial cells, macrophages, M2 macrophages, and M1 macrophages themselves **(** **Figure 7H** **)**. Also, HAVCR2 of M1 and M2 macrophages seemed to be regulated by LGALS9 of many cells, such as neoplastic cells, microglial cells, and neural stem cells. As for the growth factors, IGF2 of unknown cells showed robust correlations with IGF1R, IGF2R, and INSER of the other six cell types **(** **Figure 7I** **)**. Macrophages may interact with microgial cells *via* CCL4, and interact with T cells and oligodendrocyte progentior cells *via* IL 1B **(** **Figure 7J** **)**. The correlation with other genes could be found in **Figure 7K** by the same means.

### Differences in cells neighboring SOX10-expressed cells

**Figure 8:**
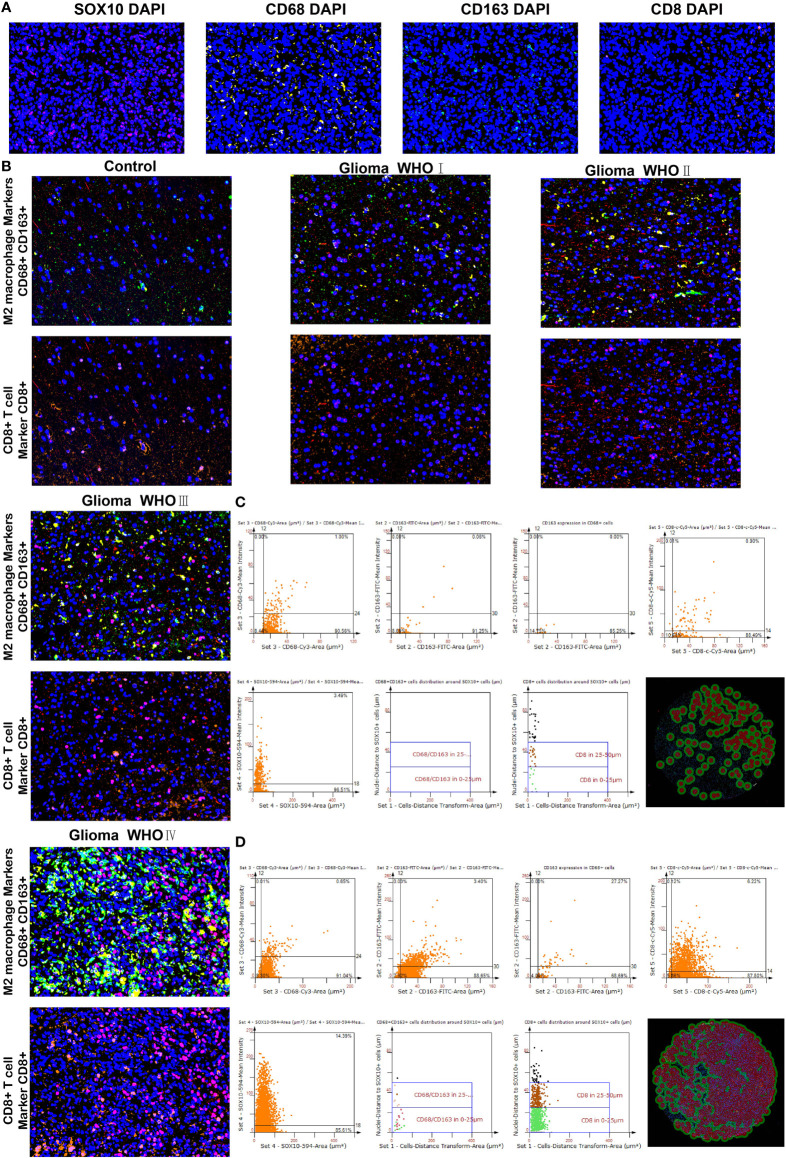
Differences in CD68, CD163 macrophages, and CD8 T cells neighboring SOX10-expressed cells. **(A)** Multiplex immunofluorescence staining of SOX10 (pink), CD68 macrophages (yellow), CD163 macrophages (green), CD8 T cells (orange), and DAPI (blue). **(B)** Multiplex immunofluorescence images of M2 macrophage markers CD68+ and CD163+, and CD8+ T cell marker CD8+ in control, Glioma WHO I, Glioma WHO II, Glioma WHO III, and Glioma WHO IV groups, respectively. The scatter diagrams display the quantity of CD68+ cells, CD163+ cells, CD68+CD163+ cells, CD8+ cells, and SOX10 expressed cells and the quantity of CD68⁺CD163⁺ cells along with CD8+ cells at different locations neighboring SOX10 expressed cells at 0–25 and 25–50 μm. Images of automatically identified staining by the TissueFAXS and StrataQuest software in **(C)** Glioma WHO III group and **(D)** Glioma WHO IV group.

We performed multiplex immunofluorescence in the controlled group and different grades of glioma groups to further characterize the relationship between SOX10-expressed cells and neighboring CD68+CD163+ cells, and CD8+ cells. The results revealed that SOX10 expression is elevated with the increase in glioma grades **(** **Figures 8A, B** **)**. Besides, with the increase in SOX10 expression, neighboring CD68+ cells, CD163+ cells, and CD8+ cells are also increased **(** **Figures 8C, D** **)**. The quantity of CD8+ cells at the distance of 0–25 μm and 25–50 μm neighboring SOX10-expressed cells exploded in the Glioma WHO IV group, while the amount of CD68+CD163+ cells also increased. Hence, we concluded that CD68+CD163+ M2 macrophages, and CD8+ T cells, were the prepotent infiltrated immune cell types in glioma. Meanwhile, SOX10 expression is a regulator of neighboring immune cells.

## Discussion

In recent years, studies revolving around gliomas are mostly concentrating on the TME, which is suggested to be one of the main obstacles to improving the prognosis and OS of HGG patients (27). To explore and clarify the mechanism of how infiltrating immune cells in the glioma TME influence the prognosis and OS, much large-scale bioinformatic analyses have been performed, and several biomarkers have been found (5, 28, 29). However, as a typical transcription factor family, the expression of the SOX family in gliomas has not been fully discussed before. We are the first to evaluate the cluster ability thoroughly and other characteristics of the SOX family and analyze SOX10 expression profiles in gliomas in prognostic potential, immune response, and co-expression in single-cell sequencing. Significantly, our results suggest that the SOX family has two distinct clusters regarding gliomas. Compared with cluster1, cluster2 seems to have a higher OS but weaker immune response. Correspondingly, the genomic profiles of the two clusters are dissimilar. IDH has co-occurrent relations with many other genes, such as ATRX, CIC, and FUBP1, which are potent regulators of cell growth (30). IDH1 is more likely to mutate in cluster2. More importantly, our analysis of SOX10 expression files in gliomas implies its predictive ability. Moreover, overexpression of SOX10 indicates a worse OS and prognosis. Also, SOX10 has the potential to predict immunotherapy response and immune effector molecules.

A complex range of genomic alterations also has clinical implications for glioma classification and prognosis. In SNV analysis, several frequent somatic mutations in gliomas, including IDH1, TP53, and ATRX, have been found to present more in cluster2 than in cluster1 (31, 32). Besides, as mentioned before, the TME has been reported to influence the gene expression of gliomas and the infiltration circumstance of stromal and immune cells, which are significant indicators of predicting prognosis (33). Data on the ESTIMATE algorithm show that stromal, immune, and ESTIMATE scores are higher in cluster1. The results indicate a worse prognosis and shorter OS in cluster1. Consequently, the SOX family is thought to have the cluster ability in gliomas to predict malignancy.

The SOX family has been confirmed to be closely associated with the immune features of the TME. In gliomas, the copious SOX family has played a crucial role in cell differentiation. Also, the SOX family and their mRNA expression levels have been associated with glioma patients’ prognosis (13). In our study, the high expression of SOX10 is related to shorter OS in glioma.

An epigenome profiling of GBM indicates that SOX10, an oligodendrocyte forerunner marker and chromatin modifier, is a dominant regulator in RTK I-subtype tumors (34). It also affects the glioma TME. This is consistent with our results. Numerous types of immune cells are enriched in high-SOX10-expression patients in our analysis of infiltrating immune cell populations. Our results suggest that an increased expression of SOX10 is associated with the DNA replication, mismatch pair, and regulation of negative regulatory T-cell differentiation. With increasing SOX10 expression, B cells, T cell CD4, and macrophages are elevated. We can infer that SOX10 is correlated with T-cell dysfunction with the heatmap. As a consequence, we draw a conclusion that SOX10 is a significant regulator in the glioma TME.

Based on the types of function and activation, macrophages can be divided into two types: M1 macrophages and M2 macrophages (35). M1 macrophages are induced by LPS, INF-γ, and TNF-α and mainly release TNF-α, CXCL9, and CCL4. M2 macrophages mainly release TGF-β and CCL1 (36). Macrophages, especially M2 macrophages, are negatively associated with the survival of glioma patients (37). In our study, macrophages elevate in the SOX10 high expression group. Especially for CD68+CD163+ M2 macrophages neighboring SOX10-expressed cells, an increased number of these macrophages are found with the elevation of SOX10 expression. Besides, CD8+ T cells are also found to explode at the distance of 0–25- and 25–50-μm neighboring SOX10 high-expression cells. The results indicate that SOX10 regulates the types and quantity of glioma infiltrated immune cells.

GSVA shows a negative association with SOX10 and cardiolipin biosynthesis, citric acid cycle, trans-sulfuration, pyrimidine metabolism, and ubiquinone. SOX10 also has pleiotropic effects in cancer-immune cascade steps. Considering metabolism is a vital determining factor in the survivability and potency of immune cells, SOX10 is supposed to be a more remarkable biomarker in immunotherapy response than B clonality (26).

The SOX family has also been found to have the ability to regulate stem and progenitor cells in adult tissues (38). Our single-cell sequencing results reveal that SOX10 is highly expressed in OPC and NPC, indicating a regulatory function. An immune checkpoint, manifesting the capability of inhibiting T-cell function, refers to specified molecular interactions at the interface between antigen-presenting cells and T cells (39). In melanoma, regulated by fat mass and obesity-associated protein, enrichment of SOX10 decreases the effect on anti-PD-1 blockade immunotherapy (40). Similarly, our data imply that SOX10 can predict anti-PD1 and anti-CTLA4 immune therapy responses. Besides, we have found correlations between SOX10 and HAVCR2, LGALS9, and CD70. These results suggest a coordinated role with SOX10 and those immune checkpoints in glioma development.

Glioma invasion is driven by autocrine signaling transmitted by secretory factors that signal through receptors on the tumor, including growth factors and cytokines (41). We have found that IGF2R, INSR, and IGF1R have a tight relationship with SOX10 in gliomas. Besides, EGFR amplification and PTEN inactivation in GBM have recently been shown to regulate the activity of the DNA repair (42). Overexpression of EGFR drives GBM cell invasion. Gefitinib is a tyrosine kinase inhibitor targeting EGFR (43). The semi-inhibition rate demonstrates that the estimated IC50 is lower in the high-expression SOX10 group than in the counterpart, which suggests that high-expression SOX10 has higher sensitivity to gefitinib; in other words, gefitinib is more effective in gliomas overexpressing SOX10 (44). Our data indicate that gefitinib might be a molecularly targeted agent for treating patients with highly expressed SOX10.

Notwithstanding, the complete comprehensive information, specific functions, and clarified mechanisms of these SOX families in gliomas and many other diseases have not been fully explored and explained. It is reported that as an oncogene, more than 50% of the cancer patients present NOTCH activation mutations (45). The activation of NOTCH significantly favors tumor progression (46). It is accordant with our data. We have discovered through the enrichment analysis that the high expression of SOX10 has a positive correlation with the NOTCH signaling pathway. Tumor-associated macrophages have a complex interaction with glioma progression (47). In our study, positive regulation of macrophage chemotaxis and activation are also related to the high expression of SOX10, which may be the reason for the elevation of macrophages in the high expression SOX10 group. Therefore, it can be surmised that the overexpression of SOX10 may activate macrophages and then elevate the number of CD68+CD163+ macrophages, which are important components of the immune microenvironment. Then, macrophages release cytokines to regulate the signaling pathway, such as NOTCH, to affect glioma progression. Consequently, we infer that the overexpression of SOX10 can promote glioma progression.

In conclusion, our study demonstrates the outstanding cluster ability of the SOX family. Cluster2 has a better prognosis and longer OS than cluster1. Concentrating on SOX10, multiple results imply that it has a multifaceted prognostic value in gliomas. In gliomas, SOX10 overexpression corresponds to immune infiltration and bleak prognosis. However, Gefitinib and Nilotinib have more utility in patients with highly expressed SOX10. Except for PD1 and EGFR, our results suggest that the high expression of SOX10 may also correlate with other potential immune checkpoints. Given that, SOX10 has the potential to be an auspicious target for glioma immunotherapy.

## Data availability statement

The original contributions presented in the study are included in the article/**Supplementary Material**. Further inquiries can be directed to the corresponding authors.

## Author contributions

Conception and design: GX and QC; Foundation support: QC, GX, and RP; Acquisition and analysis of data: GX, KW, ZW, ZD, XL, WY, and ZL; Interpretation of data: PL and JZ; Drafting the manuscript and revising for submission quality: GX, KW, and QC; All authors; Study supervision: QC and RP. All authors contributed to the article and approved the submitted version.

## Funding

Financial support was provided by the National Natural Science Foundation of China (Nos. 82171347, 82073893, and 81901268), Hunan Provincial Natural Science Foundation of China (Nos. 2022JJ30971 and 2022JJ20095), Hunan Provincial Health Committee Foundation of China (No. 202204044869), and Xiangya Hospital Central South University postdoctoral foundation.

## Acknowledgments

The author express gratitude to the public databases, websites, and softwares used in the paper. We are grateful to the High Performance Computing Center of Central South University for partial support of this work.

## Conflict of interest

The authors declare that the research was conducted in the absence of any commercial or financial relationships that could be construed as a potential conflict of interest.

The reviewer DW declared a shared parent affiliation with the authors PL and JZ to the handling editor at the time of the review.

## Publisher’s note

All claims expressed in this article are solely those of the authors and do not necessarily represent those of their affiliated organizations, or those of the publisher, the editors and the reviewers. Any product that may be evaluated in this article, or claim that may be made by its manufacturer, is not guaranteed or endorsed by the publisher.

## Supplementary material

Supplementary Figure 1
**(A)** Correlation between SOX10 and various immunotherapy drugs. Semi-inhibition rate of different SOX10 expressions in **(B)** Gefitinib and **(C)** Nilotinib.
