# Supplementary figures and images for: Machine learning-based identification of SOX10 as an immune regulator of macrophage in gliomas
Source: Front Immunol. 2022 Nov 29;13:1007461. doi: 10.3389/fimmu.2022.1007461 (PMC9745112; doi:10.3389/fimmu.2022.1007461)

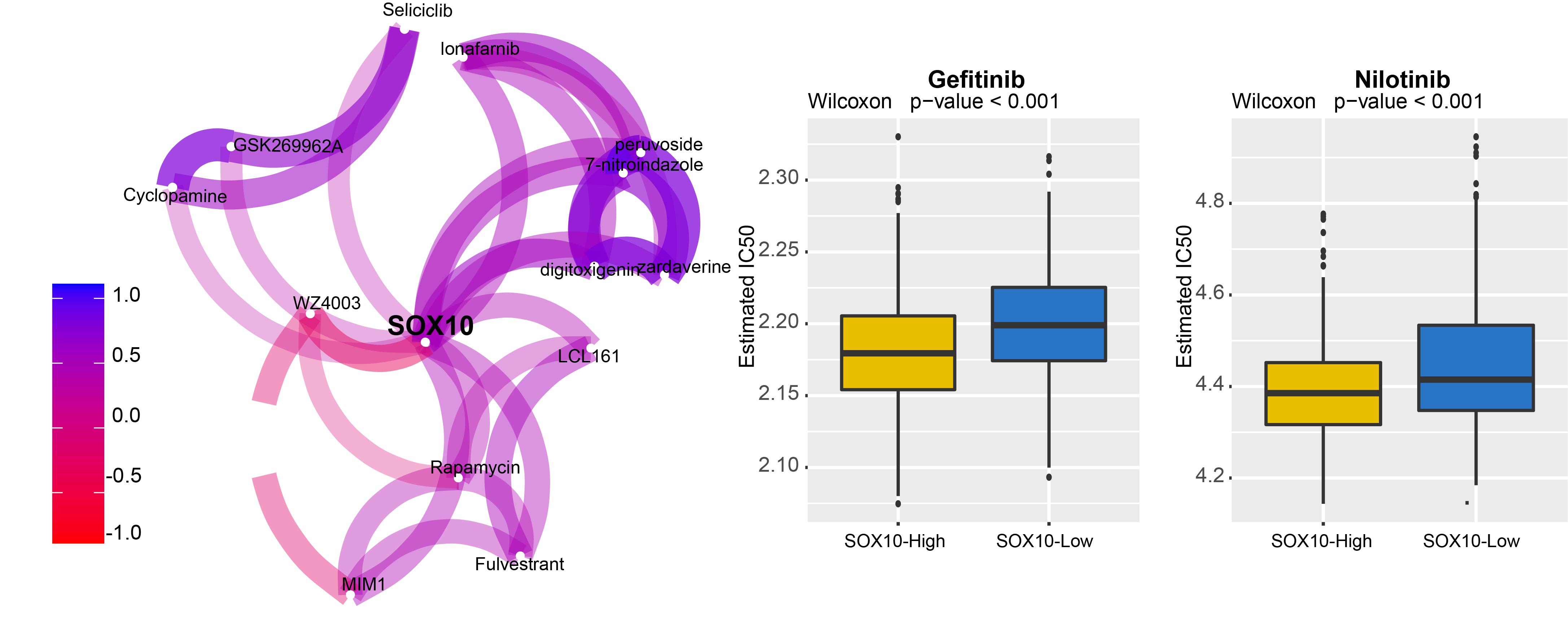

Supplement: Supplementary Figure 1 — (A) Correlation between SOX10 and various immunotherapy drugs. Semi-inhibition rate of different SOX10 expressions in (B) Gefitinib and (C) Nilotinib. [file Image_1.jpg]
